# Effect of intravenous immunoglobulin (IVIg) on primate complement-dependent cytotoxicity of genetically engineered pig cells: relevance to clinical xenotransplantation

**DOI:** 10.1038/s41598-020-68505-1

**Published:** 2020-07-16

**Authors:** Takayuki Yamamoto, Yehua Cui, Diyan Patel, Abhijit Jagdale, Hayato Iwase, David Ayares, David K. C. Cooper, Hidetaka Hara

**Affiliations:** 1 Xenotransplantation Program, Department of Surgery, University of Alabama at Birmingham (UAB), LHRB752, 701 19th Street South, Birmingham, AL 35294 USA; 2 Second Affiliated Hospital, University of South China, Hengyang, Hunan China; 3 Revivicor, Blacksburg, VA USA

**Keywords:** Preclinical research, Drug development

## Abstract

Triple-knockout (TKO) pigs may be ideal sources of organs for clinical xenotransplantation because many humans have no preformed antibody to TKO pig cells. Intravenous immunoglobulin (IVIg) is widely used for severe infection or the treatment/prevention of antibody-mediated rejection in allotransplantation. Anti-pig antibodies in IVIg could be harmful in clinical xenotransplantation. It is unknown whether anti-TKO pig antibodies are present in IVIg. The main aim of this study was to investigate in vitro whether IVIg contains anti-TKO pig antibodies with cytotoxic effect to pig cells. Undiluted pooled human serum (HS) and five different commercial preparations of IVIg were tested for IgM and IgG binding to red blood cells (RBCs) from wild-type (WT), α1,3-galactosyltransferase gene-knockout (GTKO), and TKO pigs by flow cytometry. Complement-dependent lysis of IVIg against these pig pRBCs was measured by hemolytic assay. Pooled HS and 4 of 5 IVIg commercial preparations contained anti-pig IgG that bound to WT and GTKO pRBCs, but not to TKO pRBCs. One preparation of IVIg contained antibodies that bound to TKO pRBCs, but there was no cytotoxicity of IVIg to TKO pRBCs. The results suggest that IVIg administration to human recipients of TKO pig grafts would be safe. However, the specific preparation of IVIg would need to be screened before its administration.

## Introduction

Genetically-modified pigs could be an alternative source of organs for clinical transplantation. Pig kidney or heart graft survival in nonhuman primates (NHPs) now extends to many months or even years^1–6^. However, antibody-mediated xenograft rejection and the development of a thrombotic microangiopathy in the graft remain problematic^7^. Preformed and induced antibodies directed toward the donor vascular endothelium are considered to be the primary causative factor in the development of antibody-mediated rejection, which is believed to result from activation of the vascular endothelium, mediated by antibody and/or complement activation^8^.

Intravenous immunoglobulin (IVIg) preparations are known to have immunomodulatory effects on inflammatory and autoimmune diseases^9,10^, and are used to treat severe infection^11^ and/or to prevent/treat rejection of HLA-incompatible and ABO-incompatible kidney allografts^12–14^. IVIgs are purified IgG products prepared from a pool of 5,000–10,000 blood donors, and typically contain > 95% unmodified IgG, and only trace amounts of IgA and IgM^13^. There are ten different brands of commercially-available IVIg in the United States (Table1).

**Table 1:** FDA-approved commercial IVIg products.

IVIg products	BIVIGAM	CARIMUNE	PRIVIGEN^a^	FLEBOGAMMA^a^	GAMUNEX-C^a^	GAMMAGARD LIQUID^a^	GAMMAGARD S/D	GAMMAPLEX	OCTAGAM^a^	PANZYGA
Company	Biotest Pharmaceuticals Corporation	CSL Behring AG		Grifols, Inc		Baxter Healthcare Corporation		Bio Products Laboratory	OCTAPHARMA Pharmazeutika Produktionsges	

From FDA U.S Food & Drug administration https://ww.fda.go.

^a^Used in the present study (with lot number): FLEBOGAMMA (Lot # A4GKB00121), GAMMAGARD LIQUID (Lot# LE12S081AB), PRIVIGEN (Lot# P100005816), OCTAGAM (Lot# M808A8441), and GAMUNEX-C (Lot# B3GHA00023).

IVIg can affect both innate and adaptive immunity^10,14^. The administration of IVIg has been reported to (i) delay rejection of guinea pig-to-rat heart xenotransplants (in which both species express galactose-α1,3-galactose [Gal] antigens) through anti-complement activity and/or anti-idiotypic antibodies^15^, (ii) delay rejection of wild-type (WT) pig hearts in NHPs^16^, and (iii) prolong survival of WT pig kidneys perfused with human blood ex vivo^17^.

However, low-dose (0.5 mg/kg) IVIg administration did not reduce the level of anti-Gal antibodies in HLA-sensitized patients^18^. Furthermore, IVIg has been reported to contain antibodies to Gal and to *N*-glycolylneuraminic acid (Neu5Gc)^19,20^. IVIg may also suppress the inflammatory response to a pig xenograft^10^. IVIg (i) inhibits complement-mediated inflammation by inhibition of C5b-9 and membrane attack complex^10,21^; (ii) inhibits cytokine production, e.g., IL-1β, IFN-γ, IL-2, and IL-6^10,22,23^, and (iii) induces anti-inflammatory cytokines, e.g., IL-10^10,24^. IVIg can also inhibit ischemia–reperfusion injury because IVIg is a powerful scavenger of C3b produced in the ischemic brain ^21^. Although there is considerable evidence of efficacy of IVIg (as above), anti-pig antibodies in IVIg could potentially be detrimental in clinical xenotransplantation^5^.

Pigs are now available in which expression of all three known carbohydrate xenoantigens against which humans have natural (preformed) xenoantibodies [Gal, Neu5Gc, and Sda (the product of the enzyme, β-1,4 *N*-acetylgalactosaminyltransferase)], have been deleted, i.e., triple-knockout (TKO) pigs. It is unclear whether IVIg contains any antibodies that might have an adverse effect on TKO pig xenograft survival.

The major aims of the present study were to investigate (i) whether IVIg contains antibodies against WT, α1,3-galactosyltransferase gene-knockout (GTKO), or TKO pig red blood cells (RBCs), and (ii) whether IVIg has cytotoxicity against WT, GTKO, or TKO pRBCs. We also investigated whether IVIg can reduce human serum IgG/IgM antibody binding to pig cells in vitro and in vivo*,* and whether it can inhibit human serum cytotoxicity to pig cells in vitro and in vivo*.*

## Materials and methods

### Human serum and RBC donors

Pooled human serum (pooled from 50–150 donors) was purchased from Innovative Research, Novi, MI. Whole blood was collected from two healthy human volunteers with no history of previous exposure to alloantigens (i.e., no previous pregnancies, blood transfusions, or organ transplants). The use of human blood (RBCs and serum) and collection of human blood was approved by the Institutional Review Board (IRB) of the University of Alabama at Birmingham (UAB) (#300001924). All methods for collection of human blood were carried out in accordance with relevant guidelines and regulations/Declaration of Helsinki. Participants gave informed consent per the guidelines of the IRB of UAB (#300001924). The blood was centrifuged at 910 × *g* for 5 min, the serum was separated, and stored at – 80 ˚C to retain complement activity. When required, decomplementation was carried out by heat-inactivation for 30 min at 56 °C.

### IVIg

Because only five preparations of IVIg were available through the UAB hospital pharmacy, only five brands were studied (Table1).

### Sources of pig cells

Blood was obtained from WT, GTKO, and TKO pigs (all provided by Revivicor, Blacksburg, VA), all of blood type non-A (O). Pig aortic endothelial cells (pAECs) were collected from WT and GTKO pigs. (pAECs from TKO pigs were not available to us.)

### Detection of expression of xenoantigens on pig cells by flow cytometry

pRBCs and pAECs were stained for expression of Gal (by isolectin BSI-B4), Neu5GC (chicken anti-Neu5GC antibody), and Sd^a^ (Dolichos biflorus agglutinin, DBA), as previously described^25^.

### Baboons

Baboons (*Papio* spp) from the Division of Animal Resources of the Michale E Keeling Primate Center, MD Anderson Cancer Center, Bastrop, TX, 3 years-old, weighing 7–10 kg, were used in this study. Protocols for baboon studies were approved by the Institutional Animal Care and Use Committees at the University of Alabama at Birmingham (#20673). All animal care procedures were in accordance with the *Principles of Laboratory Animal Care* formulated by the National Society for Medical Research and the *Guide for the Care and Use of Laboratory Animals* prepared by the Institute of Laboratory Animal Resources and published by the National Institutes of Health (NIH Publication No. 86-23, revised 1985).

### Isolation of RBCs and pAECs

RBCs from humans and pigs were separated from blood, as previously described^29,30^. Briefly, blood was washed × 3 with phosphate-buffered saline (PBS, Invitrogen, Carlsbad, CA), and centrifuged for 5 min at 4 °C at 910 *g*. The washed RBCs were suspended in (i) fluorescence-activated cell sorting (FACS) buffer [PBS containing 1% bovine serum albumin (BSA) and 0.1% NaNH_3_] for surface staining, or (ii) diluted to 10 × 10^6^/ml (1 × 10^6^/100 µl) in PBS at room temperature for IgM and IgG binding, or (iii) diluted to 800 × 10^6^/ml in PBS at room temperature for complement-dependent hemolytic assay.

pAECs were isolated from fresh pig aortas and cultured, as previously described^26–28^.

### Identification of RBC and pAEC surface expression of Gal, Neu5Gc, and Sd^a^

RBCs were diluted to 1 × 10^6^ cells per tube in FACS buffer. pAECs were diluted to 1 × 10^5^ cells per tube in FACS buffer. Surface expression of Gal, Neu5Gc, and Sd^a^ antigens was detected by flow cytometry, as previously described^26,28,29^.

### In vitro binding of IgG/IgM in human serum and IVIg to pRBCs and pAECs

IgG/IgM binding to pRBCs was measured after their exposure to (i) pooled human serum, and (ii) several concentrations of different IVIgs (5 preparations), as described^26,30^. IgG/IgM binding to pAECs was measured after exposure to pooled human serum + /− several concentrations of one IVIg preparation (FLEBOGAMMA), as described^26,28^.

#### pRBCs

Briefly (i) 10% (for WT and GTKO pRBCs) or 20% (for TKO pRBCs) heat-inactivated serum, (ii) titrated IVIg, or (iii) PBS (negative control) was incubated with 1 × 10^6^ RBCs for 2 h at 4 °C. To prevent nonspecific binding, after washing 10 µL of heat-inactivated goat serum was added. IgM or IgG binding was detected by incubating with fluorescein isothiocyanate (FITC)–conjugated goat anti-human IgM (mµ chain-specific^30^, polyclonal) or IgG (γ chain-specific, polyclonal) at 1:50 dilution (Invitrogen by Thermo Fisher Scientific, Eugene, OR) for 30 min in the dark at 4 °C.

#### pAECs

Briefly, 20% heat-inactivated human serum, or serial concentration of heat-inactivated IVIg (FLEBOGAMMA), or PBS (control) was incubated with 1 × 10^5^ pAECs for 2 h at 4 °C^28^. To prevent nonspecific binding, after washing 10 µL of heat-inactivated goat serum was placed in 100 µL staining buffer for 20 min at 4 °C, and was incubated with 10 µL FITC-conjugated goat anti-human IgM (mµ chain-specific^30^, polyclonal) or IgG (γ chain-specific, polyclonal) at 1:50 dilution (Invitrogen) for 30 min in the dark at 4 °C.

Data acquisition was performed with a flow cytometer (BD LSRII, BD Biosciences), and binding was measured by the relative geometric mean (rGM) value, which was calculated as follows: rGM = each GM/GM in negative control. The representative figure was showed in Supplementary Fig. 1. Each experiment was performed × 3.

### Competitive binding to pRBCs or pAECs of IVIg with IgG and IgM from pooled human serum

In order to evaluate the effect of suppressing antibody binding (i.e., idiotype) in IVIg, we incubated 10% heat-inactivated pooled human serum with titrated IVIg (FLEBOGAMMA) at room temperature for 30 min. Serum alone or serum + IVIg was then added to 1 × 10^6^ pRBCs or 1 × 10^5^ pAECs. In order to eliminate the effect of dilution, an adjustment was made using 5% sorbitol so that the final volumes were equal (Supplementary Fig. 2). IgG/IgM binding to pRBCs or pAECs was measured after exposure to pooled human serum combined with several concentrations of IVIg (FLEBOGAMMA). Each experiment was performed × 3.

### Antibody-dependent complement-mediated hemolytic assay

RBCs (800 × 10^6^/ml in 75 µl) were isolated and placed in 5 ml round-bottom tubes. In order to evaluate both the effect of suppressing antibody binding (i.e., idiotype) and anti-complement effect in IVIg, of titrated non-heat-inactivated human or baboon serum (i.e. complement activity + ; 450 µl) with PBS and/or titrated IVIg (0-360 µl) (FLEBOGAMMA) and 5% sorbitol (15–375 µl) was added to each tube (total 900 µl) and incubated at 37 °C for 150min^31^. When serum and IVIg were co-cultured titrated, IVIg was added instead of 5% sorbitol, which is a constituent solution of FLEBOGAMMA. Control samples consisted of PBS (instead of the RBC solution) + 450 µl of titrated non-heat-inactivated serum with PBS + 5% sorbitol (instead of titrated IVIg). After centrifugation at 910 g for 5 min, the supernatant was collected, and each 300 µl was transferred into 96-well plates. The released hemoglobin was measured at an optical density (OD) of 541 nm using a SpectraMax M2e plate reader (Molecular Devices Corp. Sunnyvale, CA)^32^. Data were obtained in duplicate.

Cytotoxicity was calculated, as follows:

$$\% {\text{ cytototoxicity }} = \, \left( {\left[ {{\text{A}} - {\text{C}}} \right]/\left[ {{\text{B}} - {\text{C}}} \right]} \right) \, \times {1}00,$$

where A represented the experimental value (OD), B was maximal hemolysis (OD) which was determined using human or baboon serum and 10% Triton (Sigma-Aldrich, St Louis, MO), and C was minimal (control) hemolysis (i.e., hemolysis in the absence of RBCs). Each experiment was performed × 3.

### Antibody-dependent complement-mediated cytotoxicity of IVIg (alone) to pRBCs

RBCs (800 × 10^6^ /ml in 75 µl) were isolated and placed in 5 ml round-bottom tubes. Titrated non-heat-inactivated IVIg (0–360 µl) (FLEBOGAMMA) and 5% sorbitol (15–375 µl) were added to each tube (total 450 µl), and incubated at 37 °C for 150 min to evaluate the cytotoxicity of IVIg alone [Supplementary Fig. 3(a)]. In order to evaluate the effect of exogenous complement, we added rabbit complement at 40% of the final concentration to samples, and incubated at 37 °C for 150 min [Supplementary Fig. 3(b)]. In order to evaluate the effect of soluble factors in IVIg, titrated non-heat-inactivated IVIg (0-360 µl) (FLEBOGAMMA) and 5% sorbitol were added to each tube [RBCs (800 × 10^6^/ml in 75 µl)] and incubated at 37 °C for 30 min to complete binding of IVIg to pRBCs. These samples were then washed with PBS to eliminate the soluble factors in the IVIg. Then the samples were incubated with rabbit complement (i.e., exogenous complement) at 40% of the final concentration at 37 °C for 150 min to evaluate the cytotoxicity of IVIg without soluble factors [Supplementary Fig. 3(c)]. Control samples consisted of (i) pRBCs only (spontaneous), and (ii) pRBC + sorbitol (without IVIg) + rabbit complement (negative control).

Cytotoxicity was calculated, as follows:

$$\% {\text{ cytototoxicity }} = \, \left( {\left[ {{\text{A}} - {\text{C}}} \right]/\left[ {{\text{B}} - {\text{C}}} \right]} \right) \, \times {1}00,$$

where A represented the experimental value (OD), B was maximal hemolysis (OD) which was determined using IVIg and 10% Triton (Sigma-Aldrich), and C was the negative control [see above (i) for Supplementary Fig. 3(a) or (ii) for Supplemetary Fig. 3(b, c)]. Each experiment was performed × 3.

### Antibody-dependent complement-mediated cytotoxicity (CDC) of pAECs

Cytotoxicity of pAECs was carried out using LIVE/DEAD Fixable Dead Cell Stain Kits (Invitrogen, Thermo Fisher Scientific, Eugene, OR), according to the manufacturer`s instructions.

Briefly, pAECs were diluted to 5 × 10^4^ cells per tube in medium. 50 µl (5 × 10^4^ pAECs) were added to 5 ml round-bottom tubes. Then 50 µl of decomplemented serum was added to each tube and incubated at 37 °C for 30 min. After washing with PBS, medium, or serial concentrations of IVIG (FLEBOGAMMA), or 5% sorbitol (control), and rabbit complement (final concentration 20%) was added and incubated at 37 °C for 3 h [non-heat-inactivated serum (i.e. complement activity +) was ideal source of complement like RBCs studies to evaluate both the effect of suppressing antibody binding (i.e., idiotype) and anti-complement effect in IVIg in this study (see above). However, in our preliminary study, we were not able to observe any cytotoxicity when non-heat-inactivated serum (i.e. complement activity +) was used for pAECs. Therefore, rabbit complement was used to evaluate only anti-complement effect IVIg in this assay]. After washing with PBS, 0.5 µl of fluorescent-reactive dye solution was added to 1 ml of the cell suspension with PBS, and incubated for 30 min in the dark at 4 °C. After washing with FACS buffer, 200 µl of three times diluted Fixation Buffer (BD Biosciences, San Diego, CA) was added and incubated for 20 min at 4 °C. Data acquisition was performed with a flow cytometer (BD LSRII, BD Biosciences).

Cytotoxicity was calculated, as follows:

$$\% {\text{ cytototoxicity }} = \, \left( {\left[ {{\text{A}} - {\text{C}}} \right]/\left[ {{\text{B}} - {\text{C}}} \right]} \right) \times {1}00$$

where A represented the percentage of dead cells (pAECs incubated with serum and/or IVIg and complement), B was the maximal percentage of dead cells (pAECs fixed with 70% ethanol), and C was the minimal percentage of dead cells [pAECs incubated with medium and rabbit complement only (final concentration 20%) only]. Each experiment was performed × 3.

### Binding of anti-pig IgG and IgM, hemolytic assay, and serum complement levels in a baboon administered 2 g/kg of IVIg (FLEBOGAMMA)

A naive baboon was given 2 g/kg of IVIg (equivalent to a high dose in clinical kidney allotransplantation)^13^. Sera drawn pre-IVIg (day 0), immediately after IVIg (day 0), and on days 1, 6, and 13 were collected. IgG/IgM binding to pRBCs (from WT, GTKO, and TKO pigs) was measured in vitro. A hemolytic assay, using WT, GTKO, and TKO pRBCs, was carried out. Serum C3a (classical and lectin pathways) and Bb (alternative pathway) were measured by commercial ELISA kits (C3a Plus EIA, and Bb Plus Fragment EIA, Quidel, Athens, OH). Each experiment was performed × 3.

### Statistical analysis

Continuous variables were expressed as mean + /− standard deviation (SD). Comparisons between two groups were performed using a Mann–Whitney test. Comparisons among multiple groups were performed using a Kruskal–Wallis test for continuous variables. The receiver operating characteristic (ROC) curve was used to determine cytotoxicity using RBCs cut-off points based on both the positive data (2 individual human sera against WT pRBCs) and the negative data (2 individual human serum against autologous human RBCs). The ROC curve is a graphical plot of sensitivity against 1-specificity at various discrimination cut-off points. The best cut-off point is the one that represents the best compromise between sensitivity and specificity. A p value of < 0.05 was considered statistically significant. All statistical analyses were performed using social sciences software GraphPad Prism 8 (GraphPad Software, San Diego, CA).

### Ethical approval

The use of human serum and collection of human blood was approved by the Institutional Review Board of the University of Alabama at Birmingham (#300001924). Protocols for pig and baboon studies were approved by the Institutional Animal Care and Use Committees at the University of Alabama at Birmingham (#20673).

## Results

### Expression of Gal, Neu5Gc, and Sd^a^ on pig RBCs and pAECs

WT pigs expressed Gal, Neu5Gc, and Sd^a^ antigens on their RBCs and pAECs, while GTKO pigs did not express the Gal antigen. TKO pig RBCs did not express any of the three xenoantigens on pRBCs (but pAECs were not tested) (Supplementary Figs. 4A/B). These findings were expected from previous studies^28,33,34^. WT and GTKO pAECs were positive for CD31 (vascular endothelial cell) expression (> 90%) (Supplementary Fig. 4B), as previously described^28^.

### In vitro studies with human sera

#### Comparison of pooled human serum and IVIg IgG/IgM antibody binding to WT, GTKO, and TKO pRBCs

**Figure 1:**
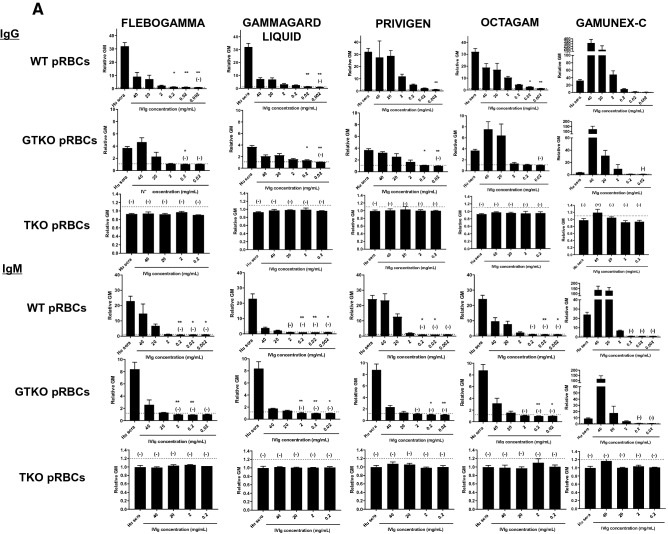

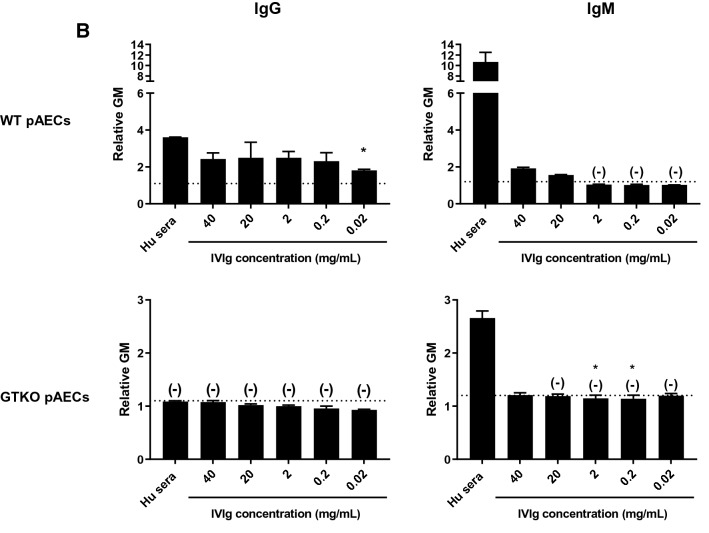
**(A)** IgG and IgM binding of pooled human serum and five different brands of commercial IVIg to WT, GTKO, and TKO pRBCs. (Note the different scales on the Y axis between WT, GTKO, and TKO pRBCs.) There was significant human serum IgG binding to WT and GTKO pRBCs, but not to TKO pRBCs. There was no IgG binding to TKO pRBCs by pooled human serum or by any IVIg, except for GAMUNEX-C. On the y axis, the dotted line represents the lowest measurable limit of binding, below which there is considered to be no binding (relative GM: IgM 1.2, IgG 1.1). Results are expressed as mean + /− SD (*p < 0.05, **p < 0.01). There was significant human serum IgM binding to WT and GTKO pRBCs, but not to TKO pRBCs. There was no IgM binding to TKO pRBCs by pooled human serum or by any IVIg. See M“Materials and methods” section (In vitro binding of IgG/IgM in human serum and IVIg to pRBCs). Heat-inactivated serum was used in this assay. On the y axis, the dotted line represents the lowest measurable limit of binding, below which there is considered to be no binding (relative GM: IgM 1.2, IgG 1.1). Results are expressed as mean + /− SD (*p < 0.05, **p < 0.01). **(B)** IgG/IgM binding of pooled human serum and IVIg (FLEBOGAMMA) to WT and GTKO pAECs. There was no difference in IgG and IgM binding to WT pAECs between pooled human serum and high-dose IVIg. There was no IgG binding of IVIg to GTKO pAECs (bottom). (TKO pAECs were not available to us.) See “Materials and methods” section (In vitro binding of IgG/IgM in human serum and IVIg to pAECs). Heat-inactivated serum was used in this assay. On the y axis, the dotted line represents the lowest measurable limit of binding, below which there is considered to be no binding (relative GM: IgM 1.2, IgG 1.1). Results are expressed as mean + /− SD (*p < 0.05).

In order to investigate whether IVIg contains anti-pig antibody (as does pooled human serum), an antibody binding assay was carried out, and binding to pRBCs was detected by flow cytometry. Six different lot numbers of pooled human serum contained no IgG or IgM that bound to TKO pRBCs, although they contained both IgG and IgM that bound to WT and GTKO pRBCs (data not shown). All 5 preparations of IVIg tested included IgG and IgM that bound to WT and GTKO pRBCS, the levels depending on the IVIg preparation (brand), but did not bind to TKO pRBCs (except for GAMUNEX-C) (Fig. 1A). When the concentration of IVIg was < 0.2 mg/ml, IgM binding to WT and GTKO pRBCs was negative. In three of the IVIg brands, however, IgG binding to WT pRBCs remained positive, even when the IVIg concentration was < 0.002 mg/ml (Fig. 1A). This result suggested that most preparations of IVIg do not contain anti-TKO pig IgG and IgM, even though all IVIg preparations contain antibodies to WT and GTKO pig cells.

#### Comparison of pooled human serum and IVIg IgG/IgM binding to WT and GTKO pAECs

In order to investigate whether IVIg contains anti-pig antibody to pAECs (similar to pRBCs), an antibody binding assay was carried out, and binding to pAECs was detected by flow cytometry. Based on the results of IgG and IgM binding to pRBCs, we selected FLEBOGAMMA, which had lower anti-nonGal pig IgG/IgM, for testing in subsequent experiments. (GAMMAGARD LIQUID became unavailable because of a shortage of supply). IgG and IgM binding (relative geometric mean [GM]) of pooled human serum and IVIg (FLEBOGAMMA) to WT and GTKO pAECs was lower than to WT and GTKO pRBCs (Fig. 1A, B). There was *no* IgG binding of IVIg to GTKO pAECs (Fig. 1B).

### Cytotoxicity of pooled human serum and IVIg to pRBCs

In order to investigate the cytotoxicity of IVIg to pRBCs, a hemolytic assay was carried out.

**Figure 2:**
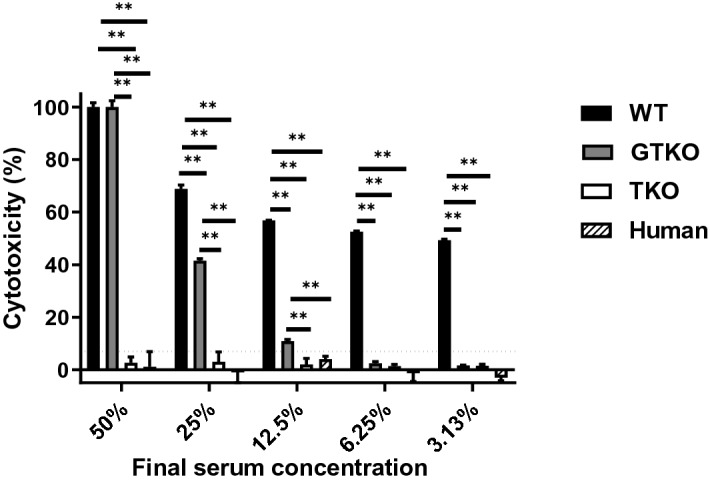
Cytotoxicity of pooled human serum against RBCs. There was no cytotoxicity of pooled human serum against TKO pRBCs or human blood type O RBCs. When the concentration of human serum was 25% or less, the cytotoxicity against GTKO pRBCs was significantly lower than that against WT pRBCs (p < 0.01). See “Materials and methods” section (Antibody-dependent complement-mediated hemolytic assay). Briefly, RBCs (800 × 10^6^/ml in 75 µl) were isolated and placed in 5 ml round-bottom tubes. Titrated non-heat-inactivated serum (i.e. with complement activity; 450 µl) with PBS and 5% sorbitol (375 µl) instead of IVIg was added to each tube (total 900 µl) and incubated at 37 °C for 150min^31^. Control samples consisted of PBS (instead of the RBC solution) + 450 µl of titrated non-heat-inactivated serum with PBS + 5% sorbitol. After centrifugation at 910 g for 5 min, the supernatant was collected, and each 300 µl was transferred into 96-well plates. The released hemoglobin was measured at an optical density (OD) of 541 nm using a SpectraMax M2e plate reader (Molecular Devices Corp). Data were obtained in duplicate. Results are expressed as mean + /− SD. The dotted line represents cut-off value (7%). (**p < 0.01).

**Figure 3:**
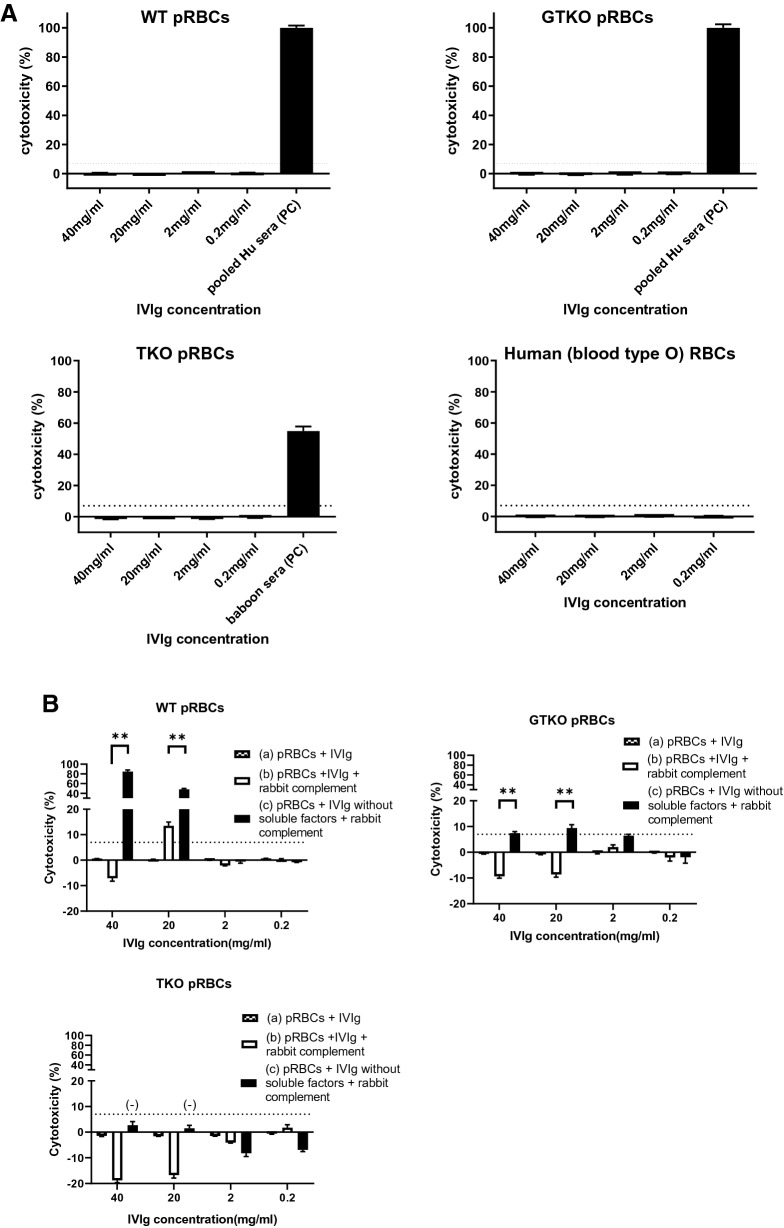

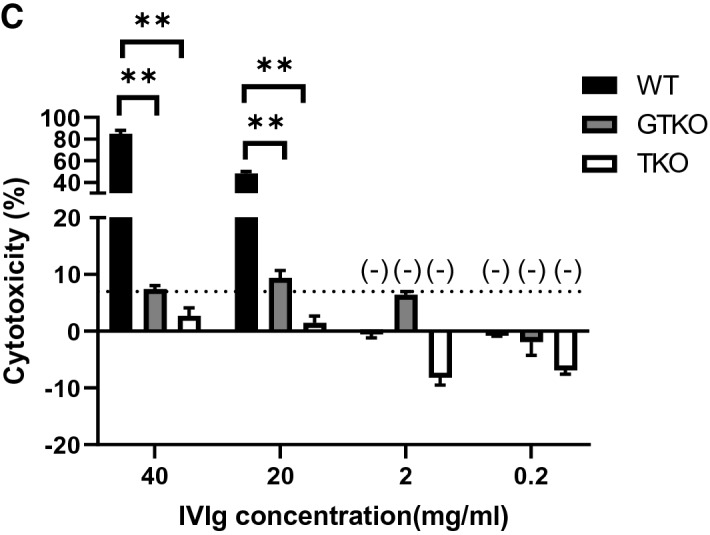
The cytotoxicty of IVIg (FLEBOGAMMA) + /− rabbit complement on the lysis of RBCs **(A)** cytotoxicity of IVIg (FLEBOGAMMA) against RBCs. The cytotoxicity of IVIg against WT, GTKO and TKO pRBCs. As a positive control (PC) for WT and GTKO pRBCs, non-heat-inactivated pooled human (Hu) serum was used. As the PC for TKO pRBCs, a naïve baboon non-heat-inactivated serum was used, because pooled Hu serum had no cytotoxicity to TKO pRBCs. Human blood type O RBCs were used as the negative control. For details of methods see “Materials and methods” section (Antibody-dependent complement-mediated cytotoxicity of IVIg alone to pRBCs). Results are expressed as mean + /− SD. The dotted line represents cut-off value (7%), below which cytotoxicity is considered negative. **(B)** Cytotoxicity of pRBCs of IVIg (FLEBOGAMMA) **(a)** without rabbit complement, **(b)** with rabbit complement, and **(c)** after washing away soluble factors from IVIg before adding rabbit complement. For details of methods see Supplementary Fig. 3 and “Materials and methods” section (Antibody-dependent complement-mediated cytotoxicity of IVIg alone to pRBCs). . When no rabbit complement was added **(a),** there was no cytotoxicity of IVIg to WT, GTKO, or TKO pRBCs. Even when adding complement **(b),** there was almost no cytotoxicity. However, after washing away soluble factors from the IVIg before adding rabbit complement **(c),** the cytotoxicity of IVIg against WT and GTKO pRBCs was significantly increased [compared to when there was no washing **(b)**] because the soluble factors in the IVIg had had a protective effect. However, the cytotoxicity of IVIg against TKO pRBCs remained negative. Results are expressed as mean + /− SD. The dotted line represents cut-off value (7%). (**p < 0.01). **(C)** Cytotoxicity associated with anti-pig antibodies in titrated IVIg (FLEBOGAMMA) against WT, GTKO, and TKO pRBCs. When the concentration of IVIg was high (> 10 mg/ml), the cytotoxicity associated with anti-WT pig antibodies was significantly higher than that associated with antibodies to GTKO or TKO pRBCs. *No* cytotoxicity was associated with the binding of anti-TKO pig antibodies in the IVIg, even though at high concentration of IVIg. Results are expressed as mean + /− SD. The dotted line represents cut-off value (7%). (**p < 0.01).

Cytotoxicity of 7% [representing the best compromise between sensitivity (1.0) and specificity (1.0)] was selected as the cut-off point for this assay, i.e., indicating no lysis. There was no cytotoxicity of human serum to human RBCs (of blood type O-negative) (Fig. 2). When the concentration of human serum was 25% or less, the cytotoxicity of human serum against GTKO pRBCs was significantly less than that against WT pRBCs (p < 0.01). There was no cytotoxicity of human serum (at any concentration) against TKO pRBCs. There was no lysis of pRBCs by IVIg alone (FLEBOGAMMA) (Fig. 3A) (even though IVIg included anti-WT and anti-GTKO pig IgG and IgM).

This result suggested that IVIg alone must decrease complement activity. We therefore measured lysis of pRBCs after the addition of 40% rabbit complement (i.e. exogenous complement) [Supplementary Fig. 3(b)]. Even when rabbit complement was added to IVIg, there was minimal or no cytotoxicity against all three types of pRBCs [Fig. 3B(b)]. However, if IVIg was allowed to bind to the pRBCs first (after IVIg and pRBCs were incubated together, followed by washing with PBS before rabbit complement was added) [Supplementary Fig. 3(c)], the cytotoxicity was significantly increased [Fig. 3B(c)], depending on the level of anti-pig IgG and IgM binding (Fig. 3C). The cytotoxicity associated with anti-WT pig antibodies in high-dose IVIg was significantly higher than that associated with anti-GTKO or anti-TKO antibodies (Fig. 3C). This result suggested that a soluble factor in IVIg might inhibit cytotoxicity. In other words, anti-pig IgG and IgM in IVIg could potentially be harmful.

### The effect of IVIg on pooled human serum IgG/IgM binding to WT and GTKO pRBCs and pAECs

In order to investigate the effect of idiotype antibody in IVIg, a competitive assay using pooled human serum, titrated IVIg, and pig cells (pRBCs or pAECs) was carried out, and the binding to pRBCs or pAECs was detected by flow cytometry.

The standard dose of IVIg used for treatment of highly HLA-sensitized recipients or in antibody-mediated allograft rejection is 1–2 g/kg as a single dose^35,36^. The increase in serum IgG in patients treated with these doses of IVIg is 10-40 mg/ml^37^. Therefore, in our experiments, we used concentrations of 40 mg/ml and 20 mg/ml (i.e., > 10 mg/ml) as high doses of IVIg.

**Figure 4:**
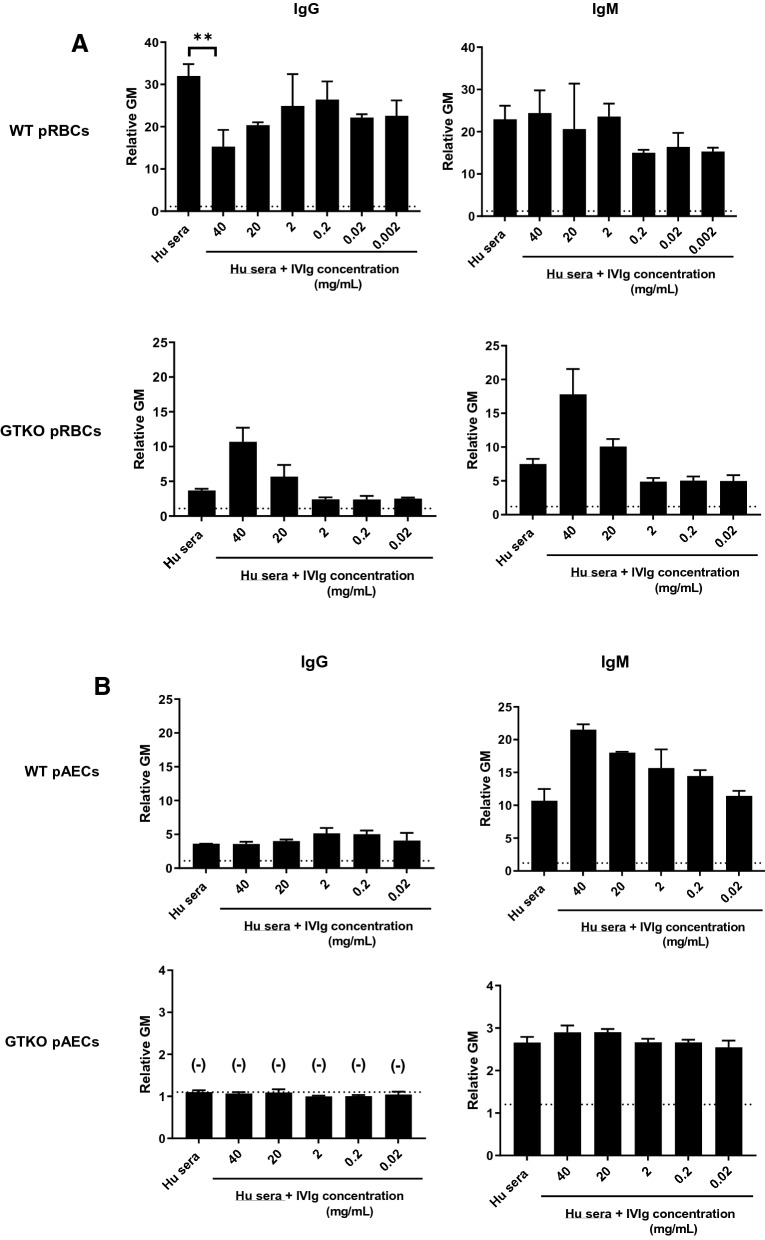
**(A)** The competitive effect of IVIg (FLEBOGAMMA) on IgG/IgM binding of pooled human serum to WT/GTKO pRBCs. High-dose (40 mg/ml) IVIg attenuated human serum IgG binding (but *not* IgM binding) to WT pRBCs (p < 0.01). IVIg increased human serum IgG binding to GTKO pRBCs, and increased IgM binding to both WT and GTKO pRBCs. See “Materials and methods” section (Competitive binding to pRBCs of IVIg with IgG and IgM from pooled human serum). Heat-inactivated serum was used in this assay. On the y axis, the dotted line represents the lowest measurable limit of binding, below which there is considered to be no binding (relative GM: IgM 1.2, IgG 1.1). Results are expressed as mean + /− SD (**p < 0.01). **(B)** The competitive effects of IVIg (FLEBOGAMMA) on IgG/IgM binding of pooled human serum to WT/GTKO pAECs. IVIg did not attenuate human serum IgG binding to either WT or GTKO pAECs and increased IgM binding. There was no IgG binding of pooled human serum or IVIg to GTKO pAECs. See “Materials and methods” section (Competitive binding to pAECs of IVIg with IgG and IgM from pooled human serum). Heat-inactivated serum was used in this assay. On the y axis, the dotted line represents the lowest measurable limit of binding, below which there is considered to be no binding (relative GM: IgM 1.2, IgG 1.1). Results are expressed as mean + /− SD.

High concentrations (40 mg/ml) of IVIg significantly inhibited human serum IgG binding to WT pRBCs (Fig. 4A), but IVIg did not inhibit IgM binding of pooled human serum to WT pRBCs, irrespective of the concentration of IVIg. These findings were expected from previous studies^15^. However, IVIg inhibited neither IgG nor IgM binding to GTKO pRBCs, but at high concentration (40 mg/ml) increased binding (Fig. 4A). IVIg did not inhibit IgG or IgM binding of pooled human serum to WT and GTKO pAECs (Fig. 4B), but at high concentrations increased IgM binding to WT pAECs.

### The effect of IVIg (FLEBOGAMMA) on the cytotoxicity of pooled human serum against pRBCs and pAECs

**Figure 5:**
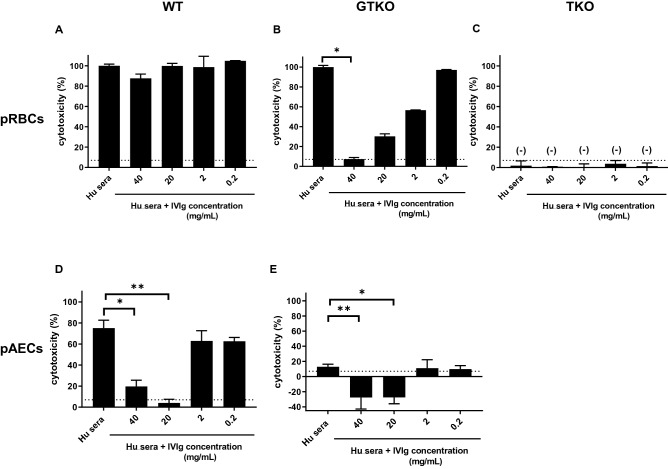
The effect of IVIg (FLEBOGAMMA) on the cytotoxicity of pooled human serum (50%) against pRBCs. **(A**) The cytotoxicity of pooled human serum (50%) against WT pRBCs was not inhibited by IVIg. **(B)** The cytotoxicity of pooled human serum (50%) against GTKO *pRBCs* was inhibited by high-dose IVIg (*p < 0.05). **(C)** There was no cytotoxicity of pooled human serum (50%) against TKO pRBCs with/without IVIg. The cytotoxicity of pooled human serum (50%) against **(D)** WT and **(E)** GTKO *pAECs* was significantly inhibited by high-dose (> 10 mg/ml) IVIg. See “Materials and methods” section [**(A)–(C)** Antibody-dependent complement-mediated hemolytic assay, and **(D, E)** Antibody-dependent complement-mediated cytotoxicity (CDC) of pAECs]. **(A)–(C)** Non-heat-inactivated serum (i.e., with complement activity) was used in the hemolytic assay. **(D, E)** Heat-inactivated serum + rabbit complement (i.e., exogenous complement) was used in the CDC assay of pAECs. The dotted line represents cut-off value (7%), below which cytotoxicity is considered negative. Results are expressed as mean + /− SD (*p < 0.05, **p < 0.01).

In order to investigate the anti-complement effect in IVIg, a complement-dependent cytotoxicity assay using pooled human serum, titrated IVIg, and pig cells (pRBCs or pAECs) was carried out. The cytotoxicity of 50% human serum against WT pRBCs was *not* inhibited by IVIg (Fig. 5A), but the cytotoxicity of 6.25% human serum was significantly inhibited by high-dose (40 and 20 mg/ml) IVIg (p < 0.01) (Supplementary Fig. 5). In contrast, the cytotoxicity of 50% and 25% human serum against GTKO pRBCs was significantly inhibited by IVIg (p < 0.05) (Fig. 5B, and Supplementary Fig. 5). These findings were anticipated from previous studies^16,38^. There was no lysis of TKO pRBCs with or without IVIg (Fig. 5C). The cytotoxicity of 50% human serum against WT (Fig. 5D) and GTKO (Fig. 5E) pAECs was significantly inhibited by high-dose (40 and 20 mg/ml) IVIg (p < 0.05), again anticipated from the studies of others^16,38^.

### The effect of IVIg (FLEBOGAMMA) on cytotoxicity of baboon sera (n = 3) against TKO pRBCs

Since there was no cytotoxicity of pooled human serum against TKO pRBCs, baboon sera (n = 3) were used to evaluate the effect of IVIg on cytotoxicity against TKO pRBCs. The cytotoxicity of 50% baboon serum to TKO pRBCs was significantly inhibited by IVIg (p < 0.05) in a dose-dependent manner (Supplementary Fig. 6).

### In vivo studies in baboons

#### Effect of the administration of 2 g/kg IVIg (FLEBOGAMMA) to a naïve baboon

To investigate the level of serum anti-pig antibodies, complement activities (C3a and Bb) and cytotoxicity to pRBCs before and after IVIg administration, 2 g/kg IVIg (FLEBOGAMMA) was administrated to a baboon.

##### IgM/IgG binding to pRBCs

**Figure 6:**
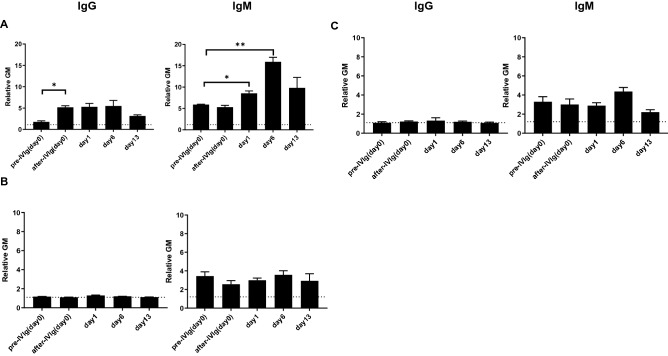
IgG and IgM binding to pRBCs after IVIg (FLEBOGAMMA) (2 g/kg) administration to a naive baboon. **(A)** In a naïve baboon, IgG binding to WT pRBCs was significantly increased after i.v. administration of 2 g/kg IVIg. IgM binding to WT pRBCs was significantly increased 1 and 6 days after IVIg. There was no significant difference in IgG or IgM binding to **(B)** GTKO, and **(C)** TKO pRBCs after i.v. administration of 2 g/kg IVIg. See “Materials and methods” section [Binding of anti-pig IgG and IgM, hemolytic assay, and serum complement levels in a baboon administered 2 g/kg of IVIg (FLEBOGAMMA)]. Heat-inactivated serum was used in this assay. On the y axis, the dotted line represents the lowest measurable limit of binding, below which there is considered to be no binding (relative GM: IgM 1.2, IgG 1.1). Results are expressed as mean + /− SD (*p < 0.05, **p < 0.01).

The intravenous administration of 2 g/kg of IVIg to a naïve baboon significantly increased serum IgG binding to WT pRBCs initially after infusion. Binding of IgM was also increased, but this did not reach significance immediately. These findings correlated with the results of previous studies^39,40^. In contrast, there was no change in serum IgG or IgM binding to GTKO, or TKO pRBCs (Fig. 6).

##### Complement activity

**Figure 7:**
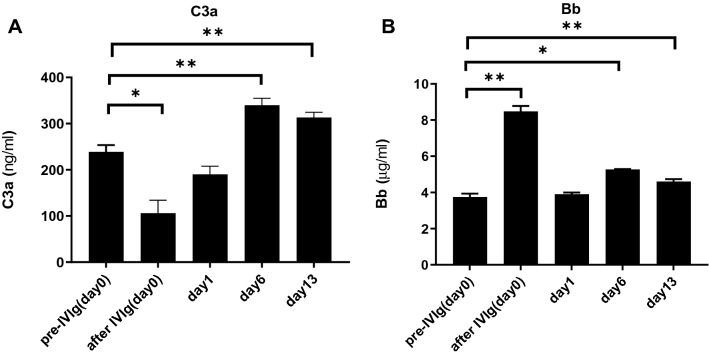
Complement activity after IVIg (FLEBOGAMMA) administration in a naïve baboon. The plasma C3a levels **(A)** immediately (day 0) after i.v. IVIg administration were significantly decreased [p < 0.05]. By days 6 and 13, however, they were significantly increased (p < 0.01). In contrast, the plasma Bb level **(B)** immediately after IVIg was significantly increased (p < 0.01). See “Materials and methods” section [Binding of anti-pig IgG and IgM, hemolytic assay, and serum complement levels in a baboon administered 2 g/kg of IVIg (FLEBOGAMMA)]. Results are expressed as mean + /− SD (*p < 0.05, **p < 0.01).

The C3a levels immediately after IVIg administration (day 0) was significantly decreased (p < 0.05). By days 6 and 13, however, the levels were significantly increased (p < 0.01) (Fig. 7). In contrast, the Bb level immediately after IVIg was significantly increased (p < 0.01), again correlating with previous reported studies^41,42^.

##### Serum cytototoxicity against pRBCs

**Figure 8:**
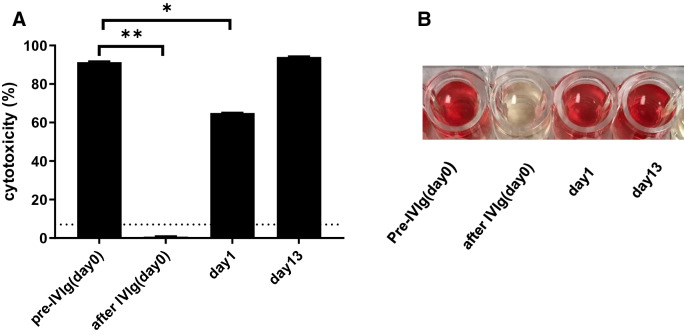
Serum cytotoxicity of baboon serum against WT pRBCs after i.v. administration of IVIg (FLEBOGAMMA) (2 g/kg). **(A)** The cytotoxicity of baboon serum (final concentration 50%) immediately (day 0) and on day 1 after IVIg (2 mg/kg) against WT pRBCs was significantly decreased. The dotted line represents cut-off value (7%), below which cytotoxicity is considered negative. See “Materials and methods” section [Binding of anti-pig IgG and IgM, hemolytic assay, and serum complement levels in a baboon administered 2 g/kg of IVIg (FLEBOGAMMA)]. Non-heat-inactivated serum (i.e. complement activity +) was used in this assay. Results are expressed as mean + /− SD (*p < 0.05, **p < 0.01). **(B)** Immediately after IVIg, lysis of WT pRBCs was completely inhibited.

As we were not able to detect any baboon serum complement-dependent cytotoxicity against GTKO or TKO pRBCs before administering IVIg, this assay was only tested against WT pRBCs (Fig. 8). The cytotoxicity of baboon serum against WT pRBCs immediately after IVIg (day 0) and on day1 was significantly decreased, correlating with previous reports^41,42^.

## Discussion

IVIg has been used for > 3 decades in allosensitized patients undergoing organ transplantation, and also in the treatment of autoimmune diseases, such as Kawasaki disease, idiopathic thrombocytopenic purpura, and myasthenia gravis^13^. In allotransplantation, there is some evidence of the efficacy of IVIg as an immunomodulatory agent in highly HLA-sensitized patients^43–45^ and in the treatment of antibody-mediated rejection^46^.

The effect of IVIg in xenotransplantation remains controversial, with some groups reporting a benefit^16,31,39,40^, but others reporting no benefit^18^ or even harm^5^. Here, we report for the first time that most preparations of IVIg do not contain anti-TKO pig IgG/IgM, and are not cytotoxic to TKO pig cells.

Anti-pig antibody levels in IVIg vary considerably depending on the brand or lot number^20^. Therefore, if IVIg is to be used in xenotransplantation, an IVIg with a low anti-pig antibody level should be selected. In our experiments, only a high-dose of IVIg had any beneficial effect.

One mechanism by which IVIg may have a beneficial effect in xenotransplantation is through the presence of anti-idiotypic antibodies against xenoreactive antibodies^15^. A second mechanism by which IVIg may have a beneficial effect is by inhibiting complement activation^10^, even though IVIg does not inhibit IgM binding to pig cells (which mediates complement activation).

IVIg contained IgM and/or IgG that bound to pRBCs and/or pAECs, but this was not associated with cytotoxicity to either cell type. Since IVIg was not associated with any cytoxicity (even when WT pRBCs were the target), we concluded that IVIg had no complement activity (Fig. 3A). When rabbit complement (i.e. exogenous complement) was added, cytotoxicity remained negative. We concluded that antibodies contained in IVIg bind to pig cells, but some other property of the IVIg inhibits cytotoxicity. In other words, the cytotoxicity to pRBCs (Fig. 5B, Supplementary Fig. 4) and pAECs (Fig. 5D, E) were significantly reduced by IVIg even though IVIg contained anti-pig (WT and GTKO) antibodies. These results suggested that the inhibition of cytotoxicity by IVIg could be associated with soluble factors in IVIg rather than by suppressing antibody binding (i.e., idiotype) to pig cells (Fig. 3B, Supplementary Fig. 3).

In our study, neither pooled human serum nor five different preparations of IVIg demonstrated any antibody binding to, or cytotoxicity against, TKO pRBCs. Treatment with IVIg, therefore, would not be detrimental if TKO pig organs were transplanted into human patients. However, after TKO pig organ transplantation into human patients, treatment with IVIg would be unlikely to have any beneficial effect unless complement is activated by the alternative pathway, e.g., as in ischemia–reperfusion injury.

IVIg might be beneficial in the treatment of serious infection and/or antibody-mediated rejection in recipients of a TKO pig organ. Moreover, if Old World NHPs, e.g., baboons or rhesus monkeys, are used as recipients (as surrogates for humans) in preclinical models of xenotransplantation, IgM and IgG binding to TKO PBMCs takes place^5^. Baboon sera (n = 3) was cytotoxic to TKO RBCs, but lysis was inhibited by IVIg (Supplementary Fig. 6). These data suggest that IVIg might provide a treatment option for antibody-mediated rejection in NHPs with TKO pig organ grafts.

In conclusion, our study suggests that most preparations of IVIg do not contain IgG or IgM directed to TKO pig cells. Therefore, it should be safe to administer IVIg to recipients with a TKO pig organ. However, the specific preparation of IVIg would need to be screened before its administration.

## Supplementary information

Supplementary Information. (PDF 423 kb)

Supplementary Legends. (DOCX 15 kb)

## Abbreviations

Gal: Galactose-α1,3-galactose
GTKO: α1,3-Galactosyltransfearse gene-knockout
IVIg: Intravenous immunoglobulin
Neu5Gc: *N*-Glycolylneuraminic acid
NHP: Nonhuman primate
pAECs: Pig aortic endothelial cells
pRBCs: Pig red blood cells
TKO: Triple gene-knockout
WT: Wild-type
