# Supplementary material for: Effect of intravenous immunoglobulin (IVIg) on primate complement-dependent cytotoxicity of genetically engineered pig cells: relevance to clinical xenotransplantation
Source: Sci Rep. 2020 Jul 16;10:11747. doi: 10.1038/s41598-020-68505-1 (PMC7367287; doi:10.1038/s41598-020-68505-1)

Supplementary Figure 1

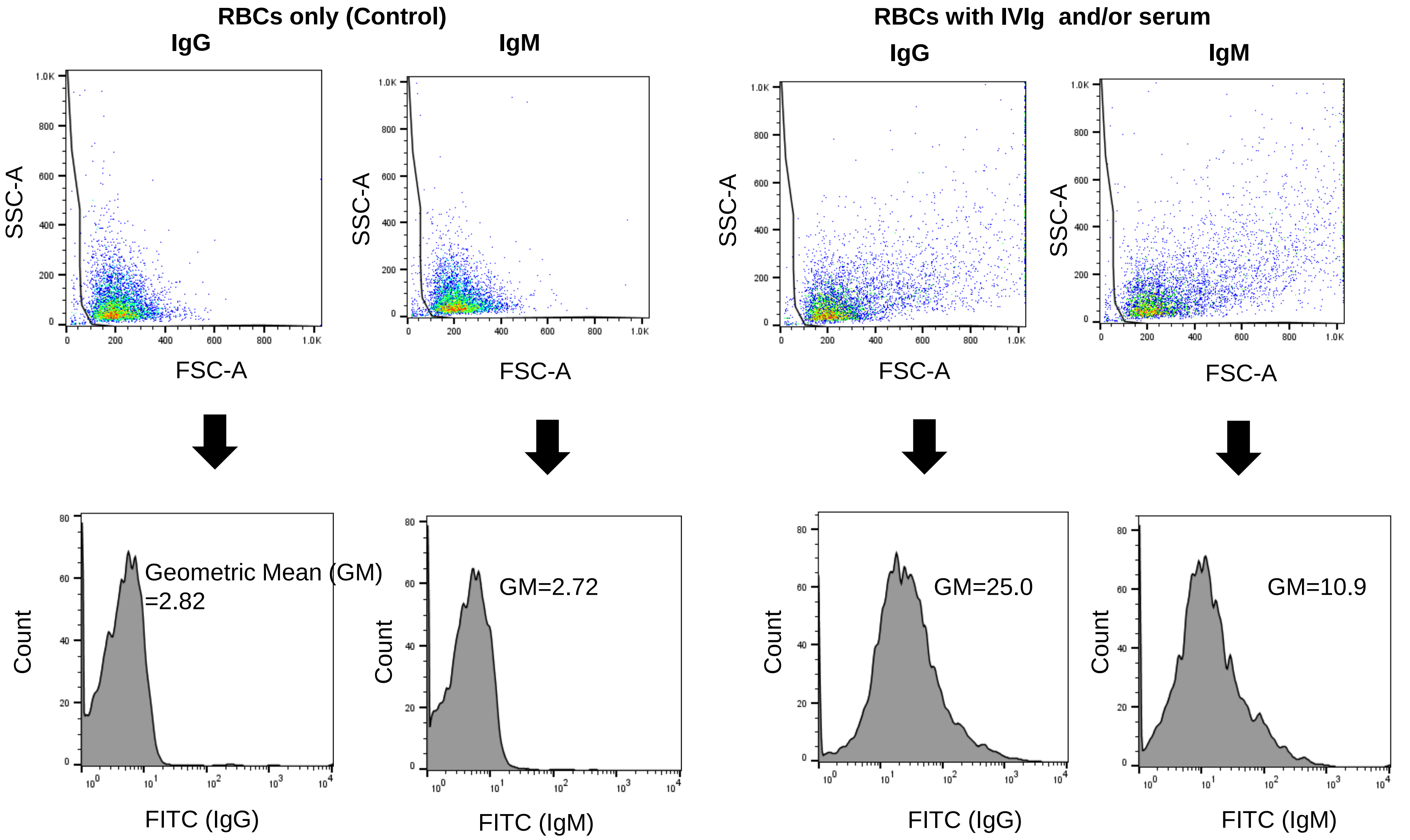

Supplementary Figure 2

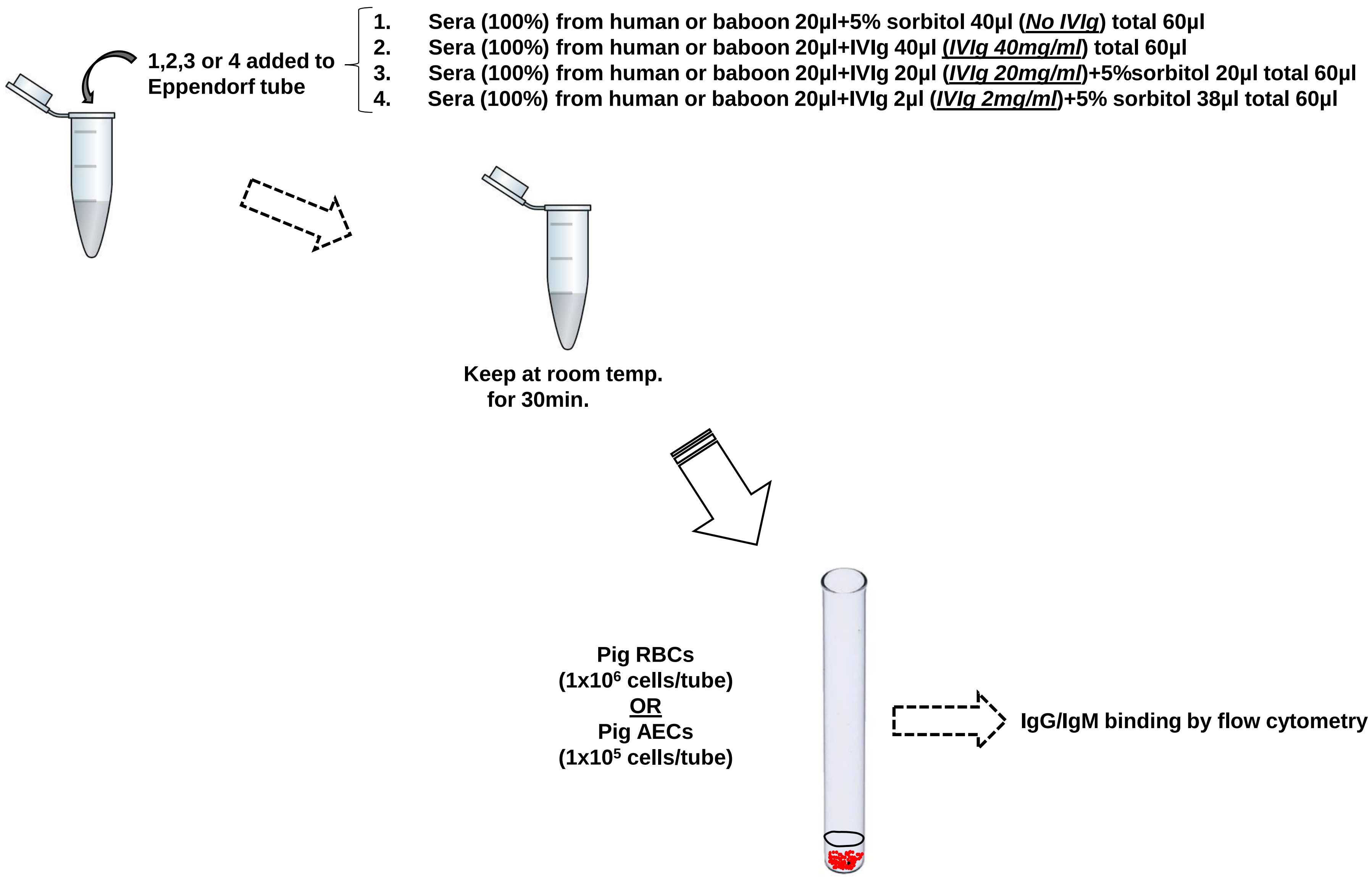

Supplementary Figure 3

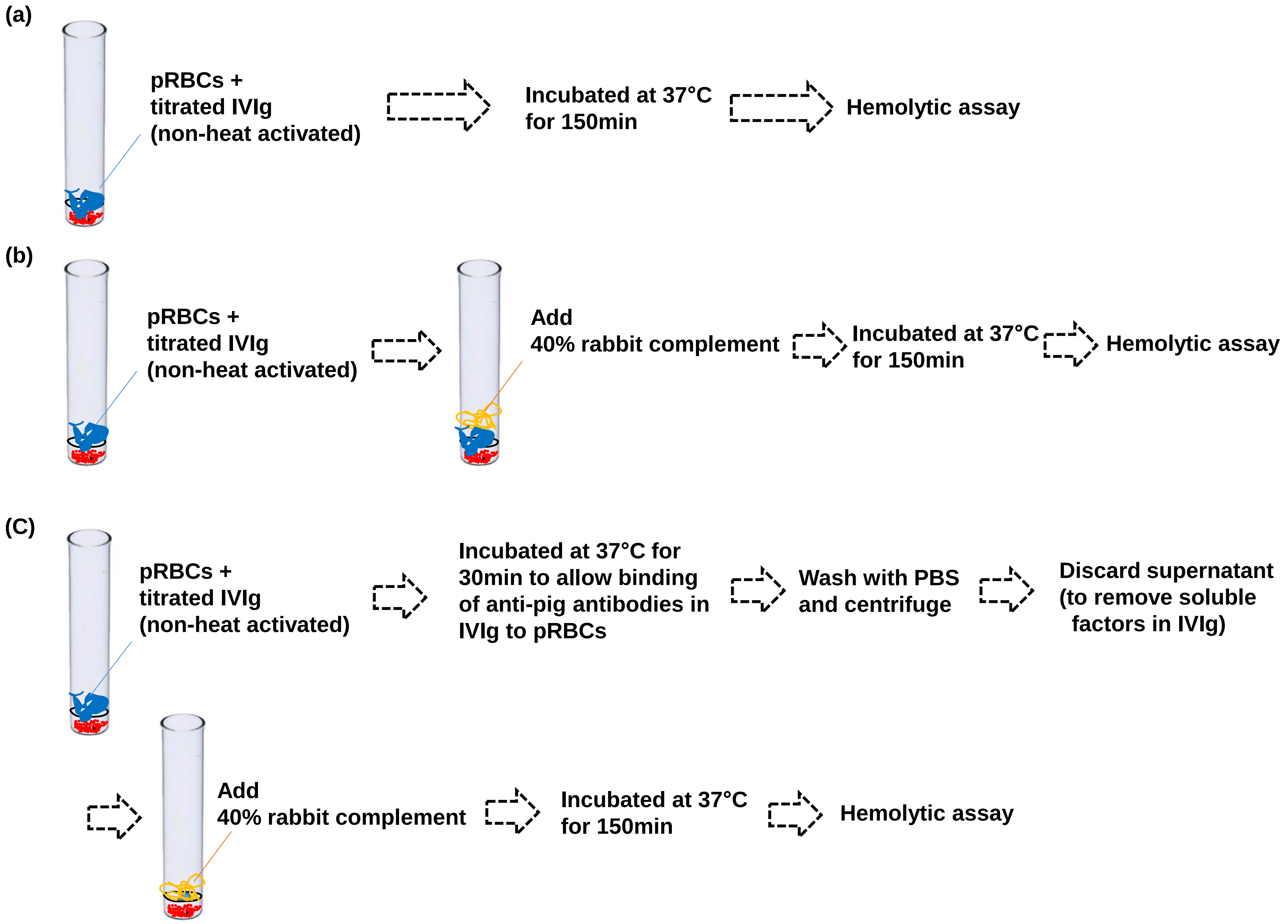

Supplementary Figure 4

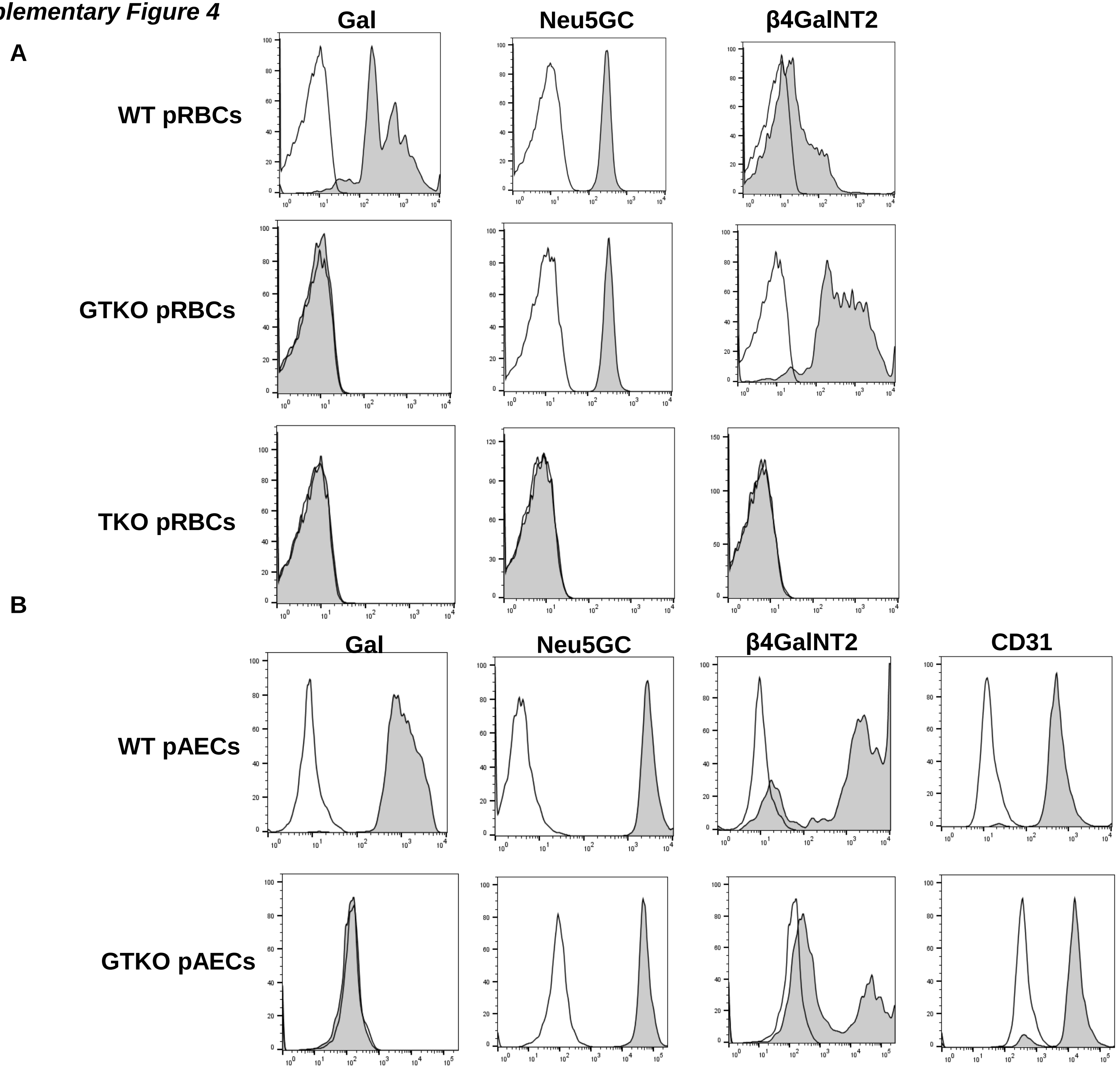

Supplementary Figure 5

WT pRBCs

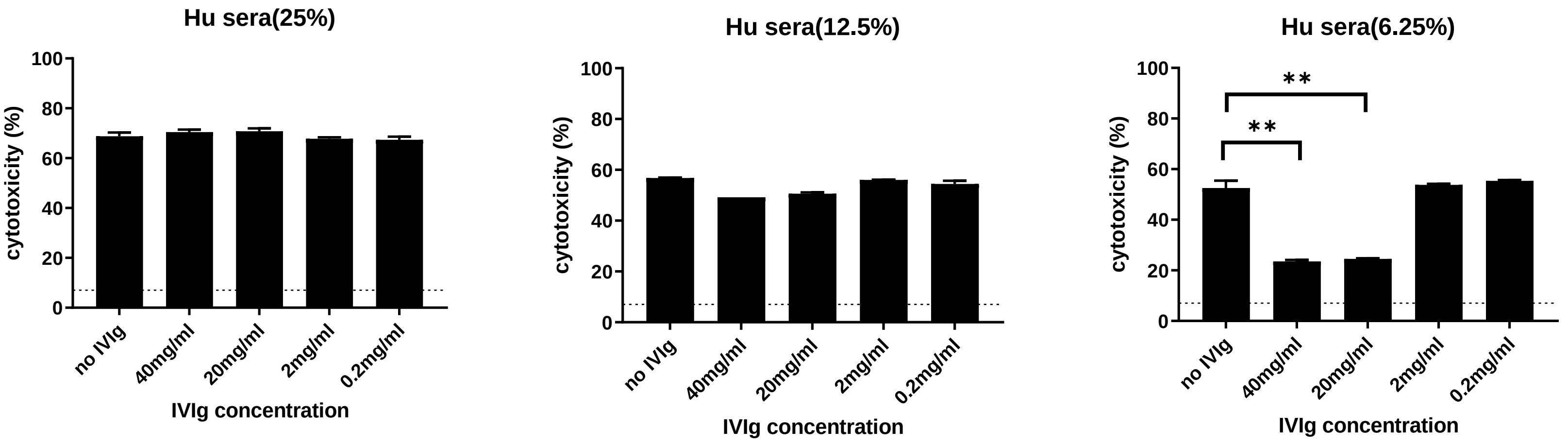

GTKO pRBCs

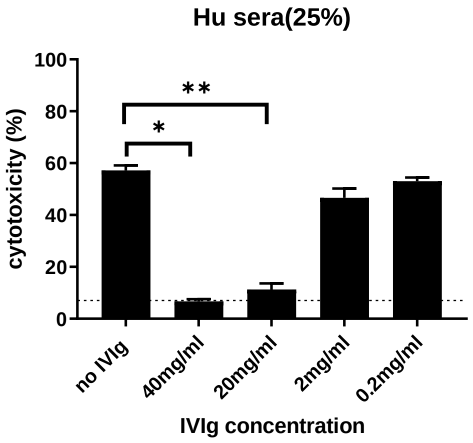

Supplementary Figure 6

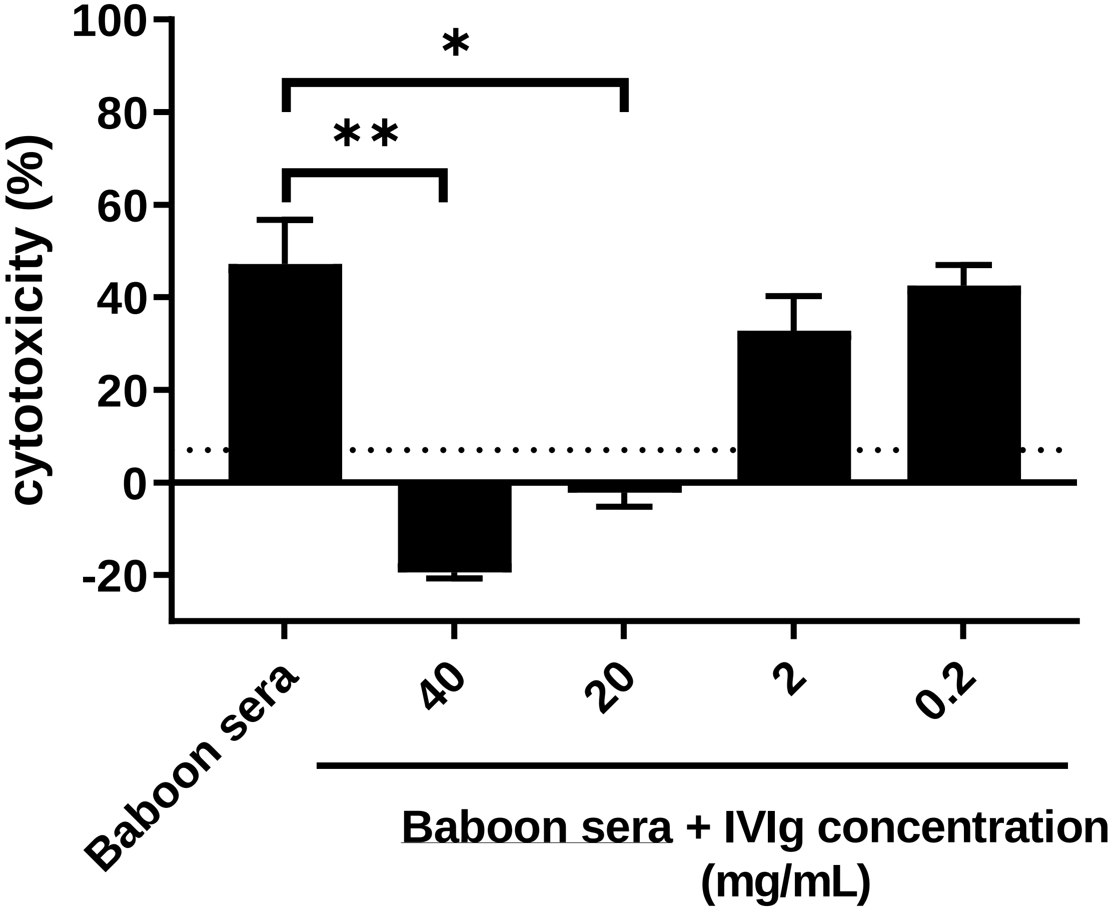

Supplement: Supplementary file 1 — Supplementary Information. (PDF 423 kb) [file 41598_2020_68505_MOESM1_ESM.pdf]
