# Supplementary material for: Effect of intravenous immunoglobulin (IVIg) on primate complement-dependent cytotoxicity of genetically engineered pig cells: relevance to clinical xenotransplantation
Source: Sci Rep. 2020 Jul 16;10:11747. doi: 10.1038/s41598-020-68505-1 (PMC7367287; doi:10.1038/s41598-020-68505-1)
Supplement: Supplementary file 2 — Supplementary Legends. (DOCX 15 kb) [file 41598_2020_68505_MOESM2_ESM.docx]

***Supplementary Figures Legends***

***Supplementary Figure 1.* Representative figure to demonstrate gating of antibody binding to RBCs.**

Forward versus side scatter (FSC vs SSC) gating was used to identify the RBCs. Left figure shows the RBCs without serum and/or IVIg as a control. Right figure shows the RBCs with serum and/or IVIg as experimental data. Binding was measured by the relative geometric mean (rGM) value, which was calculated as follows: rGM = each GM/GM in negative control.

***Supplementary Figure 2.* Method of assessing competitive binding to pRBCs or pAECs between IVIg and IgM/IgG from pooled human serum.**

***Supplementary Figure* 3. Method of hemolytic assay of WT, GTKO, and TKO pRBCs with the addition of IVIg (FLEBOGAMMA) +/- rabbit complement +/- soluble factors in IVIg.**

**(a)** Scheme of hemolytic assay of pRBCs using IVIg *without* rabbit complement. **(b)** Scheme of hemolytic assay of pRBCs using IVIg *with* rabbit complement. **(c)** Scheme of hemolytic assay of pRBCs using IVIg after removal of soluble factors in the IVIg, and then the addition of rabbit complement. Cytotoxicity of pRBCs was then measured by antibody-dependent complement-mediated hemolytic assay.

***Supplementary Figure 4.* Expression of Gal, Neu5Gc and β4GalNT2 (Sda) on WT, GTKO and TKO (GTKO/CMAHKO/ β4GalNT2KO) pRBCs (A) and expression of Gal, Neu5GC, β4GalNT2 (Sda) and CD31 on WT and GTKO pAECs (B) by flow cytometry.**

**(A)** Unstained RBCs are shown as negative controls (white). Stained RBCs are shown as gray color. WT pigs expressed Gal, Neu5Gc and Sda, and GTKO pigs expressed Neu5Gc and Sda on RBCs. TKO pigs did not express any of the 3 carbohydrates. **(B)** Unstained pAECs are shown as negative controls (white). Stained pAECs are shown as gray color. Although WT pigs expressed Gal, Neu5Gc and Sda (top) on pAECs, GTKO pigs did not expressed Gal (bottom). Both of them expressed CD31.

***Supplementary Figure 5.* The effect of IVIg (FLEBOGAMMA) on cytotoxicity of pooled human serum against pRBCs (WT and GTKO)**

The cytotoxicity of pooled human serum (25% and 12.5%) against WT pRBCs was not inhibited by IVIg. However, the cytotoxicity of human serum (6.25%) against WT pRBCs was significantly inhibited by high-dose (>10mg/ml) IVIg (p<0.01) (upper). The cytotoxicity of human serum (25%) against GTKO pRBCs was also inhibited by IVIg (p<0.05) (lower). See Materials and Methods section (Antibody-dependent complement-mediated hemolytic assay). Non-heat-inactivated serum (i.e. complement activity+) was used in the hemolytic assay. Results are expressed as mean +/- SD. The dotted line represents cut-off value (7%). *p<0.05, **p<0.01.

***Supplementary Figure 6*. The effect of IVIg (FLEBOGAMMA) on cytotoxicity of baboon sera (n=3) against TKO pRBCs**

Cytotoxicity of baboon sera (n=3) against TKO pRBCs was significantly inhibited by IVIg. See Materials and Methods section (Antibody-dependent complement-mediated hemolytic assay). Non-heat-inactivated serum (i.e. complement activity+) was used in the hemolytic assay. Results are expressed as mean +/- SD. The dotted line represents cut-off value (7%). *p<0.05, **p<0.01.
